# Potential regulators and metabolic networks of muscle fatty infiltration: genomic and radiomics investigation based on 33 300 participants

**DOI:** 10.1097/JS9.0000000000003523

**Published:** 2025-10-07

**Authors:** Jiale Tan, Zheci Ding, Jinglin Zheng, Jie Zhang, Xinyi Chen, Zhou Li, Luze Shi, Jiwu Chen, Yaying Sun

**Affiliations:** a Department of Sports Medicine, Shanghai General Hospital, Shanghai Jiao Tong University School of Medicine, Shanghai Jiao Tong University, Shanghai, China; b School of Medicine, Tongji University, Shanghai, China

**Keywords:** genetic atlas, genome-wide association study, multi-omic analysis, muscle fatty infiltration, therapeutic targets

## Abstract

**Background:**

Muscle fatty infiltration (MFI), the pathological replacement of muscle by adipose tissue in chronic diseases, lacks comprehensive genetic characterization despite known cellular contributors. Elucidating its genetic architecture and clinical correlations could reveal therapeutic targets for this debilitating condition.

**Materials and methods:**

We performed a genome-wide association study (GWAS) on 33 300 participants’ genomic/MRI data, identifying MFI-associated loci. Fine-mapping (GCTA-COJO/FUMA), Mendelian randomization (tissue-specific genes, plasma proteins, metabolites) and genetic correlation (LDSC) analyses were conducted. KLF5’s functional role was validated through inhibition experiments in fibro-adipogenic progenitors (FAPs) and murine immobilization-induced MFI models.

**Results:**

GWAS revealed 91 significant SNPs across 26 loci, with risk genes enriched in olfactory transduction and JAK-STAT pathways. Multi-omics integration identified KLF5 as a key transcriptional regulator, CHRDL2/HLA-E as circulating risk protein, and phosphatidylcholines/triglycerides as causal metabolites, and genetic correlations between MFI and metabolic/musculoskeletal disorders. Experimentally, KLF5 suppression reduced adipo-fibrogenic FAP differentiation and improved muscle histology in mice.

**Conclusion:**

Our study delineates MFI’s polygenic basis, establishes clinical-metabolic relationships, and mechanistically validates KLF5 as a target. These findings provide a framework for treating MFI through metabolic modulation or KLF5 inhibition, with broader implications for muscle-degenerative comorbidities.

**HIGHLIGHTS**

- In this study, we utilized genome and radiomic data from a large cohort to perform a genome-wide association study (GWAS) focused on muscle fatty infiltration (MFI), thereby comprehensively elucidating the genetic landscape of MFI.
- In subsequent analyses, we integrated multi-omics data, including transcriptomic and proteomic datasets, to thoroughly uncover the potential biological regulatory genes associated with MFI.
- Given the complex relationship between MFI and both local inflammation and systemic conditions highlighted in previous research, we conducted a comprehensive analysis using quantitative trait locus data derived from skeletal muscle and peripheral blood samples.
- Considering the established associations between MFI and various chronic diseases in prior studies, we leveraged the GWAS data generated in this study to investigate genetic correlations with a range of metabolism-related and musculoskeletal phenotypes. This enabled us to fully explore the role of MFI as a metabolic myopathy in influencing systemic metabolic health and muscle integrity.
- We further investigated the causal effects of various circulating lipid levels on MFI, offering novel insights that could inform clinical intervention strategies.

## Introduction

Skeletal muscle might undergo the process of fatty infiltration (MFI) under various chronic diseases, where the progressive reduction of muscle mass is inevitably replaced by the intermuscular adipose tissue^[1]^. Once the MFI was regarded merely as a consequent phenomenon of skeletal muscle degeneration; however, with accumulating evidence, researchers have found that MFI also plays an important role in the development and progression of multiple diseases, such as sarcopenia, post-rotator cuff tear muscle degeneration, etc^[2]^. Previous studies have shown the association between MFI and muscle degeneration after rotator cuff tear and the re-rupture after repair surgery^[3]^, while osteoporosis appears to be associated with increased fatty infiltration of the psoas muscle^[4]^. Otherwise, hip arthritis patients undergoing total hip replacement were found to have higher gluteal muscle fat infiltration rate than normal people^[5]^. Therefore, exploring the development mechanism of MFI and completely revealing its regulatory sites could also provide new insights for the treatment strategies of these diseases.

Extensive studies have illustrated that cells such as immune cells, circulating proteins such as IL-6 and leptin, as well as signaling pathways such as NOTCH and WNT/β-catenin, could all contribute to MFI progression in different disease models^[6,7]^. Among them, the fibro-adipogenic progenitors (FAPs), a group of mesenchymal cells that could differentiate into adipocytes that constitute MFI, were found to also play crucial roles in maintaining skeletal muscle homeostasis^[8,9]^. The FAPs could promote muscle regeneration upon acute injuries, but they could also differentiate and subsequently exacerbate muscle degeneration in chronic diseases^[10]^. In addition to specific molecular pathways and cell subsets, the relationship between MFI and the systemic metabolic state has been elucidated in previous studies. For instance, pathological metabolic processes such as insulin resistance and dysregulated lipid metabolism have been associated with abnormal fat distribution throughout the body, including MFI^[11]^. However, the studies on MFI were mainly focused on molecular mechanisms within specific cells (FAPs), which means there is still a lack of knowledge on the upstream genetic mechanism that regulates the development of MFI. Besides, most research only included limited cases on specific diseases but no further studies based on wider population were done yet.

Genome-wide association study (GWAS) tests numerous genetic variants across genomes to find the single-nucleotide polymorphism (SNP) statistically associated with a specific trait or disease^[12]^. Previous GWAS studies have been successfully applied to multiple diseases, and many replicated genomic risk loci were found to be associated with diseases, such as FTO2 for obesity and PTPN22 for autoimmune diseases, etc^[13]^. These findings unveiled important risk gene mutation sites for these diseases, providing a foundation for further in-depth exploration of gene regulation mechanisms and potential therapeutic targets. Therefore, researchers have long been hoping to conduct GWAS studies on MFI. However, the GWAS studies on MFI were limited by the lack of MRI data from large cohort, which brought certain obstacles to the exploration of MFI mechanisms.

Established in 2006, UK Biobank recruits approximately 500 000 people aged 40–69 years from 22 assessment centers across the UK^[14]^. Apart from genomic, biochemical, demographic, and socioeconomic data collected through electronic questionnaires, biological samples, and body measurements, this database also gathered MRI results of more than 30 000 participants, which finally brought hope to initiating MFI-related GWAS.

Our study performed GWAS using UK Biobank MRI data to identify MFI-associated risk loci, followed by functional annotation. We then employed multi-omics Mendelian Randomization (MR) to investigate MFI regulatory genes at both transcriptional and protein levels. Furthermore, we conducted LDSC analysis to examine genetic correlations between MFI and clinical metabolic/musculoskeletal phenotypes. Additionally, we validated the regulatory effects of several identified targets on MFI through both cellular and animal experiments, laying the foundation for the development of novel therapeutic strategies. Our work did not employ any Artificial Intelligence tools and was reported in line with the TITAN criteria^[15]^.

## Material and methods

### Data source and study population

Approval for the present study was granted by the UK Biobank (application number 95082). The MFI was sourced from the UK Biobank and assessed via Dixon MRI scans of participants’ thighs. The MFI value was determined as the mean fat infiltration in the anterior thigh muscles, which include the quadriceps femoris, sartorius, and tensor fascia latae. Additional demographic data were collected, including age, gender, the first 10 genetic principal components, and imputed genomic data. The DNAnexus platform was used to conduct quality control of UK Biobank samples based on Python. To be included in the study, participants had to meet the following criteria: their reported gender had to match their genetic sex (as determined by sex chromosomes), they had to be of white British ethnicity, and they must not have any sex chromosome aneuploidy. Participants were excluded if they were found to have excessive kinship, defined as having 10 or more third-degree relatives, or if their MFI data were missing from the 2014 assessment (instance 2). MFI value was directly used as a continuous variable in subsequent GWAS analysis.

### Ethics approval and consent to participate

All the individual data involved in the study are from the UK Biobank. The UK Biobank was approved by the North West Multi-Centre Research Ethics Committee, and all participants provided written informed consent. Specific protocol is publicly available at (http://www.ukbiobank.ac.uk/). We applied to obtain the population data related to the UK Biobank for this study. In animal procedures, this study was approved by the ethics committee of Local Animal Care Committee (approval number: AD2024060). All experiments related to animals were conducted abiding by the National Institutes of Health Guide for the Care and Use of Laboratory Animals. This study involving the acquisition of human muscle tissue samples was approved by the Ethics Committee of Shanghai General Hospital (approval number: 2022KY067).

### Genome-wide association study

Quality control of genome sequencing data was conducted using PLINK2 software version 1.0.8. SNPs were removed if they met any of the following criteria: genotype missing rate >0.1, Hardy-Weinberg equilibrium *P*-value <1e-15, minor allele frequency (MAF) <0.01, or sample missing rate >0.1. Subsequent to the quality control process, a two-step genome-wide linear mixed model association analysis was executed using REGENIE software version 2.0.0^[16]^. During REGENIE Step 1, a whole-genome regression model was applied with recommended parameters, incorporating age, sex, and principal components 1–10 as covariates. The genotypes from the UK Biobank DNA microarray were lifted to align with the GRCh37 build. In REGENIE Step 2, association analysis was conducted on imputed dosage data for a cohort of 33 300 individuals.

### Fine mapping and functional annotation of GWAS results

GCTA-COJO was utilized to perform fine mapping through a stepwise selection procedure on the chromosomes containing genomic loci^[17]^. SNPs with MAF of less than 0.01 were excluded. The collinearity exclusion threshold was set at 0.9. Additionally, SNPs were considered to be in linkage equilibrium if the distance between them exceeded 10 000 kb.

The functional annotation of SNPs identified in GWAS was performed using the FUMA website. SNPs with a *P*-value less than 5e-8 were considered genome-wide significant. Those genome-wide significant SNPs with a linkage disequilibrium *r*^2^ of less than 0.6 were defined as independent significant SNPs, while SNPs with *r*^2^ less than 0.1 were classified as lead SNPs. SNPs classified as candidate SNPs for gene mapping met the following criteria: *r*^2^ greater than or equal to 0.6 with any identified independent significant SNPs and a *P*-value less than 0.05. RegulomeDB (RDB) regulatory element scores^[18]^ and Combined Annotation Dependent Depletion (CADD) pathogenicity scores were employed to assess the potential pathogenicity and regulatory element potential of SNPs identified in GWAS. RDB score is a web-based tool utilized for analyzing the regulatory potential of non-coding genetic variants. It integrates a variety of functional genomics data, including data from ENCODE, to assess the likelihood of a genomic variant affecting gene regulation. It is a categorical score (from 1a to 7), and 1a is the highest score for SNPs with the most biological evidence to be a regulatory element. CADD score, which is computed based on 63 annotations, is a widely used metric for assessing the potential deleterious effects of genetic variants, including SNPs and small insertions or deletions. The higher the score, the more deleterious the SNP is. A threshold of 12.37 is suggested by Kicher *et al*^[19]^. Subsequently, eQTL mapping and chromosome interaction mapping were conducted based on data from GTEx (V6, V7, and V8), EyeGEx, eQTLcatalogue, eQTLGen, ENCODE, and GSE87112 in order to further confirm the regulatory relationship between these SNPs and the expression of specific genes.

After functional annotation and fine mapping of SNPs and exploring their potential regulatory effects on genes, we mapped the associated risk genes in the following ways. Positional mapping: there were candidate SNPs located in the adjacent regions of mapped genes (within 10 kb of the gene’s location) or functional consequences of candidate SNPs on these genes (including exonic, splicing, intronic, 3-UTR, 5-UTR, downstream, and upstream). eQTL and chromatin interactions mapping: there were eQTL or chromatin interactions identified between candidate SNPs and mapped genes.

### Functional enrichment analysis

Enrichment analyses of the Kyoto Encyclopedia of Genes and Genomes (KEGG) and Gene Ontology (GO) sets were conducted on the gene lists obtained from GWAS-mapped genes. Using clusterProfiler and the GOplot R package, the results of the enrichment analysis were visualized, aiding in the exploration of the molecular mechanisms underlying the connection between these genes and MFI.

### SMR analysis at transcriptome level

The eQTL data from whole blood and skeletal muscle in the GTEx database (V8) were utilized for SMR analysis using SMR1.3.1^[20]^ to identify genes whose expression is significantly associated with MFI. A gene was considered significantly related if its *P*-value was less than 0.05. HEIDI analysis was carried out to detect potential heterogeneity in SMR, with a pHEIDI of less than 0.05 indicating heterogeneity. Cis-eQTLs were excluded if they had nominal *P*-values exceeding 5E-8 or had allele frequency differences greater than 0.2 between the populations of the GWAS and eQTL summary data. Additionally, to address the effects of linkage disequilibrium (LD), an *r*^2^ threshold (default value *r*^2^ > 0.9) was used to eliminate SNPs exhibiting very strong LD with the top associated cis-eQTLs.

### MR analysis of plasma protein and lipid metabolites

Plasma protein data were extracted from the UK Biobank Pharma Proteomics Project^[20]^, originating from the plasma proteomic profiles of 54 219 UK Biobank participants and 2940 proteins, while the GWAS outcome data of blood lipid metabolites were sourced from the study conducted by Ottensmann *et al*^[21]^. The researchers conducted GWAS of 179 blood lipid metabolites in 7174 Finnish individuals and identified 495 genome-trait associations in 56 genetic loci, including 8 novel loci.

We selected instrumental variants (IVs) associated with the levels of plasma proteins and lipid metabolites according to the three key assumptions of Mendelian Rondomization (MR). First, a strong and statistically significant association must exist between the genetic variants and the proteins or lipids. Second, the selected IVs should not be influenced by confounding factors that might affect the relationship between the exposure and outcome. Finally, the IVs must exert their effect on MFI exclusively through the exposure, without acting through alternative pathways. Specific identification criteria of IVs were as follows: (1) SNPs at the genome-wide significance level (*P* < 5 × 10^−8^) as instrumental variables; (2) LD threshold was set as 0.01 for LD parameter (*r*^2^) and a genetic distance of 10 000 kb. The LD parameter (*r*^2^) was estimated based on 1000 Genomes European panel. In addition, when selecting the pQTLs of plasma protein, we also required the SNPs located within 1 Mb from the gene encoding the protein.

In MR analysis, four methods were employed, including inverse-variance weighted (IVW), weighted median, weighted mode, Wald ratio, and MR-Egger. Generally, IVW was set as the main analysis method. A *P*-value of <0.05 was considered indicative of a significant causal relationship. When using these thresholds to screen for SNPs, we ensured the absence of heterogeneity or horizontal pleiotropy in MR estimates, as indicated by Cochran’s *Q P*-value (>0.05), and MR-Egger intercept *P*-value (>0.05). Otherwise, the *R*^2^ and *F* statistics for each SNP were calculated by the following equations: *R*^2^ = 2 × (1 − MAF) × MAF × *β*^2^); *F* = *R*^2^ (*n* − 2)/1 − *R*^2^. SNPs with F less than 10, which indicate a high risk of bias in weak instrumental variables, were removed. Besides, leave-one-out analysis was conducted to examine whether the causal relationship between exposure and outcome was influenced by a single SNP. MR analyses were performed using TwoSampleMR in R software (4.4.1). Finally, the false discovery rate (FDR) approach was used to correct for multiple testing in proteome results.

### Bayesian colocalization analysis

We conducted Bayesian colocalization analysis to determine whether the observed associations of genes with MFI were attributable to shared causal genetic variants. This analysis evaluates five specific hypotheses. Hypothesis H0 posits that neither phenotype 1 nor phenotype 2 shows a significant association with any SNP loci in a given genomic region. Hypotheses H1 and H2 suggest that either phenotype 1 or phenotype 2 is significantly associated with SNP loci within the region. Hypothesis H3 proposes that both phenotypes exhibit significant associations with SNP loci, but these are driven by different causal variants. Finally, Hypothesis H4 asserts that both phenotypes are significantly associated with SNP loci driven by the same causal variant. For the colocalization analysis, SNPs located within a ±50 KB range upstream and downstream of the genes were included.

### Genetic correlation analysis

Using linkage disequilibrium score regression (LDSC), we assessed the genetic correlation (rg) between MFI and skeletal muscle-related and metabolic phenotypes. GWAS summary statistics were filtered based on the HapMap3 reference. We excluded non-SNP variants (such as indels), as well as SNPs that were strand-ambiguous, repeated, or had a MAF of less than 0.01. LDSC evaluates the relationship between test statistics and LD to determine whether the observed inflation arises from a genuine polygenic signal or bias. This methodology allows for the estimation of genetic correlation using GWAS summary statistics without bias introduced by sample overlap. To estimate genetic covariance, *z*-scores for each variant from Trait 1 were multiplied by *z*-scores for each variant from Trait 2, and this product was regressed on the LD score. The genetic correlation is represented by the genetic covariance normalized by SNP-heritability. Statistical significance was defined as *P* < 0.05.

### Cell culture

FAPs were obtained from Shanghai Zhong Qiao Xin Zhou Biotechnology Co. FAPs were collected based on our established protocol^[22]^. Primary antibodies included: Alexa Fluor 488-conjugated anti-mouse CD31 antibody (Biolegend, CA, United States), Alexa Fluor 488-conjugated anti-mouse CD45 antibody (Biolegend, CA, United States), APC-conjugated anti-mouse integrin α7 antibody (R&D, MN, United States), and APC-conjugated/cyanine7-conjugated anti-mouse Sca1 antibody (Biolegend, CA, United States). And FAPs were marked as CD31-CD45-integrinα7-Sca1 + cells. For proliferation, cells were cultured in DMEM (Thermo, C11995500BT) containing 20% FBS (Gibco, 10099141C), 10% horse serum (Sigma-Aldrich, H0146), 1% penicillin/streptomycin (Yeasen, 60162ES76), and 2.5 ng/L bFGF (Thermo, PHG0367) at 37°C with 5% CO₂. For adipogenic differentiation, the medium was replaced with DMEM containing 10% FBS, 0.25 μM dexamethasone, 0.5 mM IBMX, 1 μg/mL insulin, 5 μM troglitazone, and antibiotics (10 000 U/mL penicillin and 10 000 μg/mL streptomycin).

### Cell viability assay

Cell viability was evaluated using the CCK-8 assay (Beyotime). FAPs were seeded in 96-well plates (5000 cells/well) and allowed to adhere for 24 h. The medium was then replaced with fresh medium containing ML264 (MCE, HY-19994, an inhibitor of KLF5 expression that has been proven to be effective in previous studies) at varying concentrations (1, 3, 6, or 10 μmol/L). After 24 h of treatment, 10 μL of CCK-8 solution was added per well, followed by 1 h of incubation. Absorbance was measured at 450 nm using a microplate reader.

### Human skeletal muscle tissue collection and preparation

Human muscle samples were collected during anterior cruciate ligament reconstruction using autologous hamstring tendon. Immediately after surgical excision, tissue specimens were flash-frozen and stored at −80°C until processing. RNA extraction was conducted using the RNA extraction kit (Xinbei) according to the manufacturer’s instructions.

### Quantitative real-time PCR (qRT-PCR)

Total RNA in cell models was extracted from FAPs treated with 6 μmol/L ML264 for 24 h using an RNA extraction kit (Xinbei). RNA concentration of FAPs and human muscle tissues was quantified via Nanodrop (Thermo Fisher Scientific). cDNA synthesis was performed using the PrimeScript RT Reagent Kit (TaKaRa), followed by SYBR Green-based qPCR (TaKaRa) on a Bio-Rad CFX Connect Real-Time PCR system (*n* = 3). Relative mRNA expression of Klf5, COL1A1, α-SMA, CEBPA, SLC25A37, KPNA3, NUDT14, APOM, and C4B was determined using the 2^(−ΔΔCt) method, with GAPDH as the reference gene. Primer sequences are listed in Supplemental Digital Content Tables S1 and S2, available at: http://links.lww.com/JS9/F268.

### Western blotting

After treatment, FAPs were lysed in RIPA buffer (Gibco) and centrifuged at 12 000× g for 10 min at 4°C. Protein concentration was determined via BCA assay. Western blotting was performed as previously described^[23]^. Primary antibodies included anti-KLF5 (21017-1-AP, Sanying), COL1A1 (14695-1-AP, Proteintech), α-SMA (14395-1-AP, Proteintech), and CEBPA(AF6333, Affinity), with Actin (66009-1-Ig, Sanying) as the loading control. Band intensities were quantified using ImageJ (NIH), and relative expression was calculated as the ratio of target protein to Actin (*n* = 3).

### Animal experiment

Ten-week-old male C57BL/6 mice (Shanghai JieSiJie Laboratory Animal Co., Ltd, average weight 27 g) were housed under a 12-h light/dark cycle. Following anesthesia with sodium pentobarbital, mice were randomly assigned to three groups: the control group (*n* = 6), the immobilization group (*n* = 6), and the ML264-treated group (*n* = 6). The immobilization group and ML264-treated group were placed on the console as bilateral lower limbs fixed by simple plaster, and the lower limb braking model was established to simulate the disuse of skeletal muscle. In addition, before immobilization, the ML264-treated group would be injected with 50 µL of ML264 (6 μM) into the gastrocnemius muscle. The gastrocnemius muscle was collected after 7 days of immobilization under isoflurane gas anesthesia. After the gastrocnemius muscle was collected, the mice were killed by cervical dislocation under anesthesia. All animal procedures were conducted in accordance with the ARRIVE guideline^[24]^.

### Histological and immunofluorescence staining

Muscle tissues were fixed in 4% paraformaldehyde for 24 h, paraffin-embedded, and sectioned (5 μm). For H&E staining, sections were stained with hematoxylin and eosin and imaged under a microscope (Nikon). Masson’s trichrome staining was performed by sequential treatment with hematoxylin (5 min), acidic ethanol differentiation (1% HCl in 70% ethanol, 30 s), ponceau magenta, phosphomolybdic acid (5 min), and aniline blue (10 min). Myofiber cross-sectional area and collagen deposition were quantified using ImageJ. For immunofluorescence, antigen retrieval was performed in sodium citrate buffer (pH 6.0, 20 min), followed by blocking (1% BSA + 0.2% Triton X-100 in TBS, 1 h). Sections were incubated overnight (10 hours) at 4°C with primary antibodies against α-SMA (14395-1-AP, Proteintech) and PPARγ (ab209350, Abcam), then counterstained with DAPI (Solarbio). Images were acquired using a fluorescence microscope (Leica).

### Statistical analysis of experimental results

Statistical analyses were performed using SPSS software (version 19.0). Continuous variables were expressed as mean ± standard deviation and analyzed using *t*-test or one-way ANOVA followed by Bonferroni *post hoc* tests, as appropriate. *P*-value of less than 0.05 was considered statistically significant. The relative gene expression levels in skeletal muscle tissue were subjected to quadratic normalization against their respective means prior to linear correlation analysis via GraphPad Prism version (9.0.0).

## Results

### Comprehensive description of MFI by GWAS

**Figure 1.**
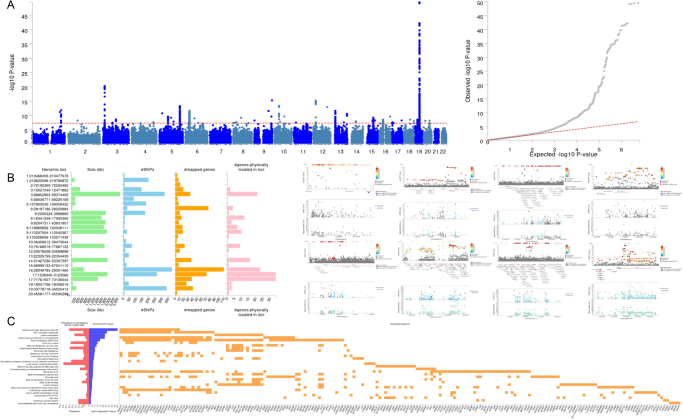
Comprehensive description of GWAS results. (A) Manhattan plot and QQ plot of GWAS analysis results. (B) Left: The characteristics of different genomic risk loci, including the regional size, the number of SNPs, and associated genes; right: locuszoom plot of genomic risk loci with the largest number of SNPs (including RDB and CADD scores of corresponding SNP sites). (C) The main related phenotype of associated risk genes has been reported in past GWAS studies.

The research design of this study is shown in graphical abstract. According to the quality control standards described in the Methods section, we excluded 469 077 participants and finally included the sample data of 33 300 participants. The GWAS identified a total of 2397 candidate SNPs (*P* < 0.05, *r*^2^ ≥ 0.6), comprising 1377 genome-wide significant (*P* < 5e-8), 91 independent significant SNPs (*P* < 5e-8, *r*^2^ < 0.6) (Supplemental Digital Content Table S3, available at: http://links.lww.com/JS9/F268), and 36 lead SNPs (*P* < 5e-8, *r*^2^ < 0.1), distributed across 26 genomic risk loci located on 16 chromosomes (Fig. 1A and B). The majority of these SNPs resided within intronic and intergenic regions. Notably, the risk locus on chromosome 19 (position: 33 896 432) harbored the highest number of genome-wide significant (*n* = 323) and independent significant (*n* = 35) SNPs.

### Fine mapping and functional annotation

For the identified genome-wide significant SNPs above, we used GCTA-COJO to conduct fine mapping analysis of these SNPs, so as to eliminate the influence of LD on the GWAS results and confirm the potential causal SNPs. Fine mapping identified 55 causal SNPs from 26 genomic risk loci (Supplemental Digital Content Table S4, available at: http://links.lww.com/JS9/F268). Subsequently, we employed RDB regulatory element scores to assess their regulatory element potential. Among which two SNPs exhibited RDB scores less than 4, indicating significant regulatory element potential, including rs17036160 (*β* = −0.1863, *P* = 5.87E-21, 2b, intronic in PPARG) and rs35283599 (*β* = 0.0834181, *P* = 6.38E-10, 2b, intronic in SMAD6). Furthermore, to detect a potential regulation of these significant SNPs on gene expression, we examined the eQTL effects and chromatin interaction effects of SNPs across different genomic risk clusters, revealing multiple genes bearing dual chromatin interaction and eQTL effects with aforementioned SNPs (Supplemental Digital Content Figure S1, available at: http://links.lww.com/JS9/F268). For the complete functional annotation results of genome-wide significant SNPs, please refer to Supplemental Digital Content files, available at: http://links.lww.com/JS9/F269.

Following the initial functional annotation of the SNPs, we determined risk genes based on the following three parameters: the number of SNPs physically located within gene regions, SNPs with eQTL associations with these genes, and the presence of chromatin interaction effects with these genes. As a consequence, several risk genes highly correlated with MFI were located, including SULT1A1 (positional mapped SNP: 70, eQTL mapped SNP: 373), PEPD (positional mapped SNP: 294, eQTL mapped SNP: 328), CCDC101 (positional mapped SNP: 63, eQTL mapped SNP: 373), and SULT1A2 (positional mapped SNP: 56, eQTL mapped SNP: 373) (complete list in Supplemental Digital Content Table S5, available at: http://links.lww.com/JS9/F268). KEGG and GO analyses elucidated biologically relevant pathways associated with MFI. KEGG enrichment analysis implicated the following pathways in potential association with MFI: Olfactory transduction, Calcium signaling pathway, JAK-STAT signaling pathway, and cAMP signaling pathway. In parallel, GO analysis identified associations with cellular divalent inorganic cation homeostasis (biological process), nuclear envelope (cellular component), and olfactory receptor activity (molecular function) (Supplemental Digital Content Figure S2, available at: http://links.lww.com/JS9/F268). In addition, we looked up GWAS catalog and found that these mapped genes were reported to be related to many diseases (asthma and major depressive disorder, autism spectrum disorder or schizophrenia, and inflammatory bowel disease), dietary habits (fish and plant-related diet, and lamb consumption), and body fat distribution in previous GWAS studies (Fig. 1C).

### Regulatory genes associated with MFI in skeletal muscle

**Figure 2.**
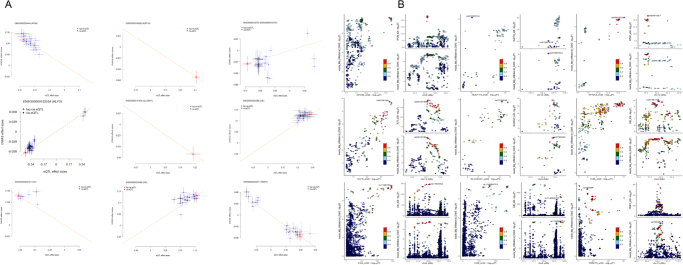
Potential regulatory genes of MFI in skeletal muscle and blood confirmed by transcriptome analysis. (A) eQTL scatter plot of the effect of transcriptome-positive genes on MFI. (B) Bayesian colocalization analysis results of transcriptome-positive genes.

To discover potential regulators of MFI, we employed SMR analysis to identify genes whose expression in muscle tissue might significantly influence MFI risk. After performing FDR multiple testing correction (FDR < 0.05) and Bayesian colocalization analysis (PPH4 > 0.8) for further validation, the expression of six protein coding genes remained significantly causally associated with MFI risk: KLF5 (*β* = 0.14, FDR = 0.01, PPH4 = 0.98), SLC25A37 (*β* = − 0.31, FDR = 0.01, PPH4 = 0.97), KPNA3 (*β* = 0.34, FDR = 0.01, PPH4 = 0.97), NUDT14 (*β* = − 0.14, FDR = 0.009, PPH4 = 0.95), APOM (*β* = − 0.37, FDR = 0.0003, PPH4 = 0.95) and C4B (*β* = 0.36, FDR = 0.005, PPH4 = 0.90) (Fig. 2).

### Regulatory genes and proteins associated with MFI in circulation

As MFI is well-established to be related to systemic status, eQTL data from peripheral blood were utilized to identify genes associated with MFI. After FDR correction (FDR < 0.05) and colocalization analysis (PPH4 > 0.8), the expression levels of five genes showed a robust causal association with MFI, including C4A (*β* = −0.18, FDR = 7.71E-05, PPH4 = 0.96), C4B (*β* = 0.11, FDR = 1.57E-07, PPH4 = 0.98), TRIM13 (*β* = −0.22, FDR = 0.03, PPH4 = 0.87) (Fig. 2).

**Figure 3.**
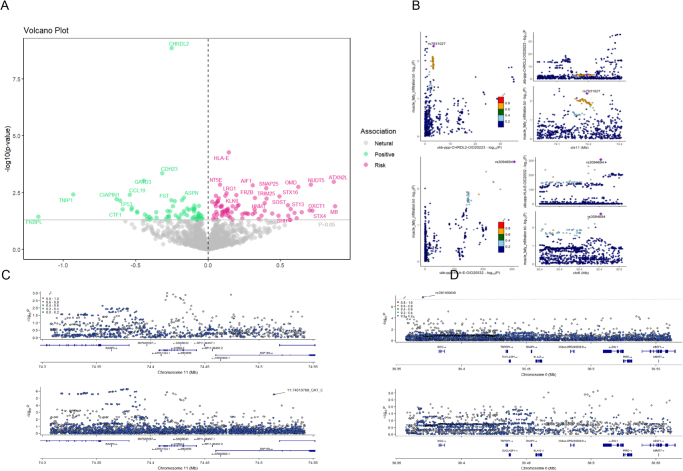
Proteomic analysis results of potential regulatory genes of MFI. (A) Volcano plot of circulating protein with significant *P*-value (<0.05) without multiple test correction. (B) Bayesian colocalization analysis results of CHRDL2 and HLA-E (circulating protein with significant effect after FDR correction). (C) Colocalization regional plot of pQTL data of CHRDL2 and MFI GWAS summary data. (D) Colocalization regional plot of pQTL data of HLA-E and MFI GWAS summary data.

Similarly, circulating protein analysis also pinpointed several candidates with causal relationships. After FDR correction, CHRDL2 (*β* = −0.25, FDR = 1.6E-6) and HLA-E (*β* = 0.14, FDR = 0.03) remained robust (Supplemental Digital Content Figure S3, available at: http://links.lww.com/JS9/F268). Colocalization analysis of HLA-E showed a PPH4 value of 0.677, below 0.8 but above 0.6, suggesting an acceptable likelihood that its circulatory pQTL shares the same genetic locus as the trait of interest (i.e. MFI). In contrast, the PPH4 for CHRDL2 was only 0.209, making it less convincing (Fig. 3).

### Interactions between MFI and musculoskeletal health

**Figure 4.**
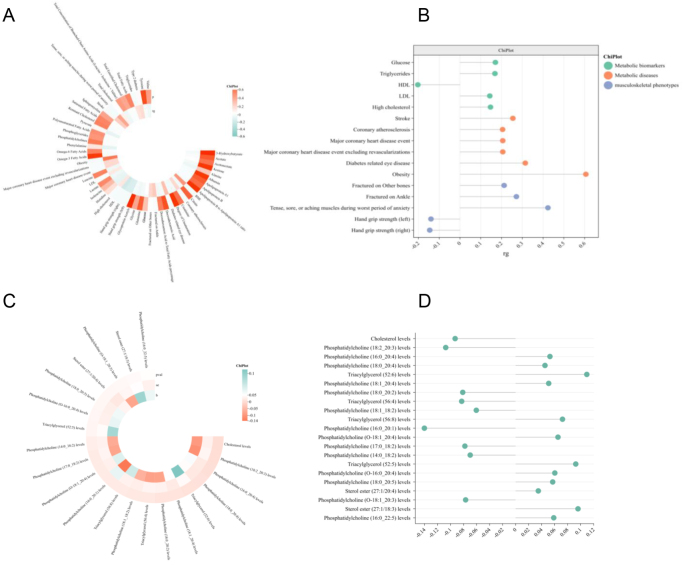
Genetic association between metabolic/musculoskeletal phenotype and MFI and causal effect of blood lipid metabolites on MFI. (A) Cluster plot of LDSC results between metabolic/musculoskeletal phenotypes and MFI. (B) Effect diagram of phenotype with significant genetic correlation with MFI, *x*-axis represented effect size (rg value), and *y*-axis showed phenotypes. (C) Cluster plot of lipid metabolites with significant causal effect with MFI. (D) Effect diagram of lipid metabolites, *x* axis represented effect size (*β* value), and *y* axis showed lipid metabolites.

LDSC analysis found a significant genetic correlation between various musculoskeletal phenotypes and MFI. Among them, hand grip strength left (rg = −0.1454, *P* < 0.0005) and hand grip strength right (rg = −0.1394, *P* < 0.001) were negatively correlated with MFI, while aching muscles (rg = 0.4234, *P* < 0.0004) and fracture in the ankle (rg = 0.2716, *P* < 0.01748) or other bones (rg = 0.2126, *P* < 0.03241) are positively correlated with MFI. Interactions between MFI and metabolic phenotypes (Fig. 4A and B).

### Interactions between MFI and metabolic phenotypes

Otherwise, the genetic association between multiple metabolism-related phenotypes and MFI was also revealed. Metabolic-related diseases such as obesity(rg = 0.606, *P* < 0.0009), diabetes related eye disease (rg = 0.3145, *P* < 0.0126), major coronary heart disease event excluding revascularizations(rg = 0.206, *P* < 0.0033), major coronary heart disease event (rg = 0.206, *P* < 0.0033), coronary atherosclerosis (rg = 0.2052, *P* < 0.0005) and stroke (rg = 0.2547, *P* < 0.002), as well as risk metabolic indexes such as low density lipoprotein (rg = 0.1436, *P* < 0.0369), triglyceride (rg = 0.1684, *P* < 7.76E-05) and glucose (rg = 0.1706, *P* < 0.0003) were positively correlated with MFI. However, HDL, a protective metabolic index, was negatively correlated with the risk of MFI (rg = −0.2027, *P* < 1.07E-05) (Fig. 4A and B).

### Lipid metabolites profile and MFI

MR analysis of 179 lipid metabolites and MFI unveiled multiple circulatory metabolites significantly influencing MFI risk (absolute beta values greater than 0.1 and *P*-values less than 0.05) (Fig. 4C and D). Two phosphatidylcholine (PC) metabolites, PC (18:2_20:3, *β* = − 0.107) and PC (16:0_20:1, *β* = −0.140), were protective against the risk of MFI, whereas Triacylglycerol (52:6) may increase the risk (*β* = 0.109). Other metabolites (such as cholesterol and sterol esters) showed statistically significant associations with MFI risk, but the small effect size prevented them from holding research value (Supplemental Digital Content Table S6, available at: http://links.lww.com/JS9/F268).

### Sensitivity analysis results in SMR and MR

All genes ultimately identified through the SMR analysis of blood and muscle transcriptomes demonstrated no significant heterogeneity in the HEIDI test, as indicated by a *P*-value greater than 0.05 (pHEIDI > 0.05). Additionally, the positive outcomes in the final validation of circulating proteome (CHRDL2 and HLA-E) and lipid metabolites (PC [18:2_20:3] and PC [16:0_20:1]) suggest the absence of significant horizontal pleiotropy or heterogeneity, as assessed in the sensitivity analysis (Supplemental Digital Content Tables S7–S8, available at: http://links.lww.com/JS9/F268). However, although the MR results of triglycerides did not show significant pleiotropy, they showed some heterogeneity.

### KLF5 expression was positively correlated with adipo/fibrogenic markers in skeletal muscle tissue

To further validate the regulatory effects of the identified candidate genes in an independent cohort, we collected 10 human skeletal muscle samples and examined the relationships between KLF5, SLC25A37, KPNA3, NUDT14, APOM, and C4B gene expression levels and adipo/fibrogenic markers (CEBPA, COL1A1, and α-SMA) using tissue qPCR. We found that KLF5 demonstrated significant positive correlations with CEBPA (*P* < 0.034, *R*^2^ = 0.378), COL1A1 (*P* < 0.01, *R*^2^ = 0.462), and α-SMA (*P* < 0.029, *R*^2^ = 0.565). In contrast, no significant associations were observed between SLC25A37, KPNA3, NUDT14, APOM, or C4B and these markers (*P* > 0.05) (Supplemental Digital Content Figure S4, available at: http://links.lww.com/JS9/F268).

### KLF5 is associated with adipogenic and fibrogenic differentiation of FAPs

Following stabilization and passaging in growth medium, FAPs were cultured in six-well plates under either adipogenic differentiation or growth conditions (*n* =3 per group). After 24 hours of culture, total RNA was extracted and subjected to qRT-PCR to examine expression differences of six potential target genes previously identified in skeletal muscle tissue analysis between differentiated and undifferentiated FAPs.

Our results demonstrated that both KLF5 and APOM exhibited significantly higher expression levels in differentiated FAPs compared to their undifferentiated counterparts (*P* < 0.01). However, while the elevated expression pattern of KLF5 aligned with its analytical association with reduced MFI risk, APOM showed an incongruent expression trend relative to its predicted effect direction in the analysis. Consequently, we selected KLF5 as the primary regulatory target for subsequent experimental validation.

**Figure 5.**
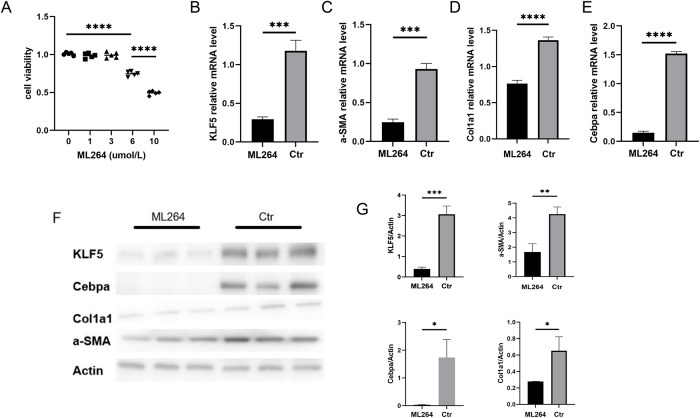
Effect of KLF5 inhibitor ML264 on adipogenic and fibrogenic ability of FAPs cells. (A) Effect of ML264 at different concentrations on the proliferation rate of FAPs cells in CCK8 experiment. (B–E) Effect of ML264 treatment on the mRNA expression level of KLF5 and fibro-adipogenic biomarkers (α-SMA, CEBPA, COL1A1) in adipogenic differentiated FAPs. (F–G) Effect of ML264 treatment on the protein level of KLF5 and fibro-adipogenic biomarkers (α-SMA, CEBPA, COL1A1) in adipogenic differentiated FAPs.

Treatment of adipogenic differentiated FAPs with ML264 revealed dose-dependent effects: CCK-8 assays demonstrated that 6 μM ML264 significantly inhibited FAP proliferation, with 10 μM exhibiting even stronger suppression (Fig. 5A). To minimize potential off-target cytotoxic effects, we employed 6 μM ML264 for subsequent experiments. Both qPCR and Western blot analyses confirmed that ML264 treatment significantly downregulated mRNA and protein expression of KLF5 along with adipogenic/fibrogenic markers (CEBPA, α-SMA, and COL1A1) (Fig. 5B–G), indicating that KLF5 inhibition attenuates the adipo-fibrogenic differentiation capacity of FAPs.

### Intramuscular ML264 administration ameliorates disuse-induced muscle atrophy, fibrosis, and fatty infiltration

**Figure 6.**
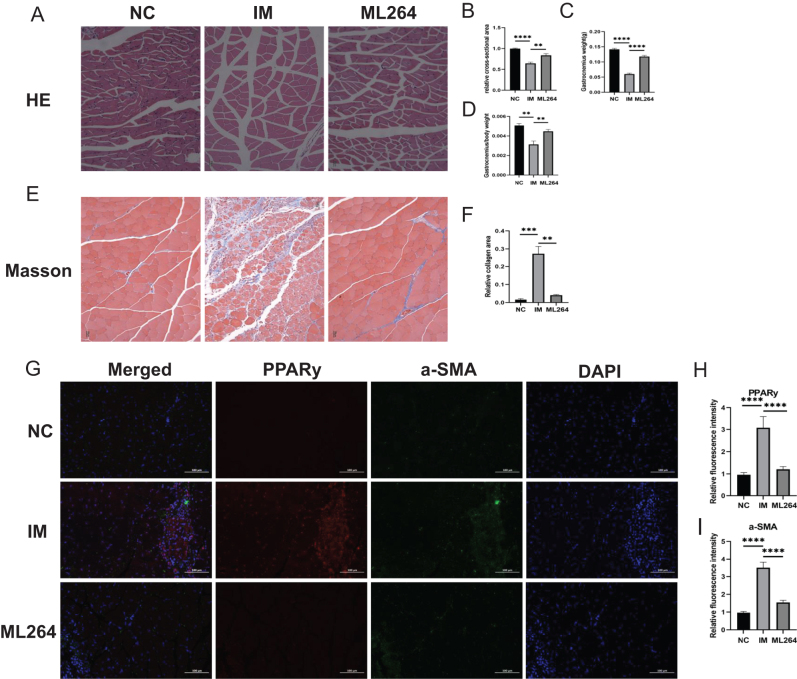
Improvement effect of ML264 intramuscular injection on disused muscle atrophy, fibrosis and fatty infiltration. (A–D) ML264 can significantly improve disuse muscular atrophy in H&E staining and muscle weight. (E–F) The relative collagen deposition area of ML264 treatment group decreased significantly in Masson staining. (G–I) The expression of adipogenic marker (PPARy) and fibroblast marker (α-SMA) in ML264 treatment group decreased significantly in immunofluorescence staining.

All experimental mice (6v 6v 6) survived through the study period without unexpected mortality and were included in the final analysis, maintaining good overall health status except for the immobilized hindlimbs. Histopathological analysis revealed that ML264 treatment substantially mitigated cast immobilization-induced muscle atrophy, as evidenced by H&E staining (*P* < 0.0029) (Fig. 6A and B) and corresponding gastrocnemius muscle weight measurements (*P* < 0.0001) (Fig. 6C and D). Masson’s trichrome staining demonstrated significantly reduced collagen deposition in ML264-treated mice compared to immobilized controls (*P* < 0.0006) (Fig. 6E and F), confirming the therapeutic effect against disuse-associated fibrosis. Immunofluorescence further showed marked downregulation of adipogenic (PPARγ) (*P* < 0.0001) and fibrogenic (α-SMA) (*P* < 0.0001) markers in ML264-treated muscle tissue (Fig. 6G–I), substantiating its dual inhibitory action on fatty infiltration and fibrotic progression.

## Discussion

Previous studies have found a genetic basis of MFI closely associated with some obesity-related genes. For instance, the FTO gene promotes MFI by disrupting the binding of the ARID5B repressor, thereby relieving the suppression of the pre-adipocyte enhancer. This leads to a doubling in the expression of IRX3 and IRX5. This altered gene expression drives the differentiation of pre-adipocytes into energy-storing white adipocytes, simultaneously inhibiting mitochondrial thermogenesis and increasing lipid deposition, which directly drives the development of MFI^[25]^. This study is the first complete multi-omics study on the genetic architecture of MFI. Using UK Biobank MRI/genomic data, we performed GWAS to identify MFI-associated SNPs, constructed a risk gene landscape through fine-mapping, and validated tissue-specific effects via SMR transcriptomic analysis. Proteomic MR revealed circulating risk proteins, while LDSC-based genetic correlation analysis linked MFI to metabolic/musculoskeletal phenotypes. MR analysis of 179 metabolic biomarkers further elucidated these associations. Furthermore, experimental validation established KLF5’s therapeutic potential in mitigating MFI and related pathologies in FAPs and immobilization models.

Initially, we conducted a GWAS to identify SNPs associated with MFI risk. Following fine mapping and functional annotation of these loci, we identified 55 potential causal SNPs across multiple chromosomes. Notably, two SNPs – rs17036160 (intronic to PPARG) and rs35283599 (intronic to SMAD6) – showed particularly strong regulatory potential based on RDB scores and demonstrated eQTL effects on their respective genes. PPARG encodes a nuclear receptor transcription factor critically involved in lipid metabolism and adipocyte differentiation. Its activation has been previously shown to promote fatty acid uptake and storage, directly contributing to intramuscular fat deposition and MFI progression^[26]^. Conversely, the SMAD6 gene encodes an inhibitory SMAD protein that acts as a negative feedback regulator in the transforming growth factor-beta (TGF-β) and bone morphogenetic protein (BMP) signaling pathways, blocking transcriptional activation of downstream genes in these pathways^[27]^. Given the roles of BMP and TGF-β in adipogenesis and muscle fibrosis, respectively^[28]^, the impact of SMAD6 on MFI risk was likely associated with its inhibitory effects on these pathways. In addition, following the procedures described in the Methods, we identified risk genes associated with MFI and performed KEGG and GO enrichment analyses for these genes. In the KEGG analysis, we found that the Olfactory transduction, Calcium signaling pathway, and JAK-STAT signaling pathway are highly correlated with MFI. Olfactory transduction initiates through the activation of G protein-coupled receptors, which stimulate adenylate cyclase, leading to elevated cAMP levels and subsequent triggering of calcium ion (Ca^2^⁺) release^[29]^. Otherwise, disruption of calcium signaling pathway can result in mitochondrial calcium overload. This overload inhibits oxidative metabolism and promotes lipogenesis (fat synthesis)^[30]^, thus olfactory transduction pathway potentially contributes to the development of MFI together with calcium signaling pathway. While the JAK-STAT signaling pathway has not been directly linked to MFI in prior studies, it has been extensively studied in the context of skeletal muscle homeostasis^[31]^. In addition, the relationship between MFI and neuromuscular junction/mitochondrial autophagy has also been partially studied. Defects in mitophagy (specifically the PINK1/Parkin pathway) lead to the accumulation of dysfunctional mitochondria. This accumulation increases reactive oxygen species (ROS) production and induces apoptosis (programmed cell death) in myofibers, thereby contributing to adipose tissue infiltration. Concurrently, neuromuscular junction dysfunction, such as disrupted aggregation of acetylcholine receptors, accelerates muscle denervation. This denervation process exhibits a positive correlation with the severity of MFI^[32–34]^. The GO analysis further confirmed several physiological processes related to IFN-γ and IL-15, as well as molecular functions such as Olfactory receptor activity. IFN-γ has been established as being closely associated with muscle regeneration^[35]^, while IL-15 has been shown to directly regulate the activity of FAPs, a key cell population in MFI^[36]^.

After preliminarily identifying risk SNPs and genes related to MFI, we further explored potential regulatory genes for MFI at the transcriptomic level using eQTL data from muscle tissues. We found that the expression of KLF5, KPNA3, and C4B genes in skeletal muscle increased the risk of MFI, while expression of SLC25A37, NUDT14, and APOM reduced this risk. While these genes have not been directly linked to MFI in previous studies, they play crucial roles in biological processes such as lipid metabolism, inflammatory response, and mitochondrial dysfunction, potentially affecting MFI via these processes. Particularly in the context of metabolic diseases such as obesity and diabetes, alterations in these genes may promote fat deposition. For instance, KLF5 (Kruppel-like Factor 5) is a transcription factor involved in cellular processes like proliferation, differentiation, and metabolic regulation^[37]^. It has been shown that KLF5 expression promotes adipogenesis through interaction with C/EBPs and PPARG, as well as influencing fat storage through metabolic pathways^[38]^. The SLC25A37 gene encodes Mitoferrin-1, an iron transporter in the mitochondrial inner membrane, essential in cellular energy and iron metabolism^[39]^. Dysfunction in SLC25A37 can lead to iron homeostasis imbalance and mitochondrial dysfunction^[40]^, which leads to mitochondrial iron overload and catalyzes lipid peroxidation. This process generates ROS and promotes the accumulation of lipid peroxides, ultimately inducing lipid deposition in both hepatic and muscular tissues. Simultaneously, iron overload exacerbates MFI through upregulation of lipogenic genes via the HIF-1α/SREBP1c pathway^[41,42]^. Following the identification of potential regulatory genes at the transcriptional level in skeletal muscle tissue, we identified KLF5 as a key regulator of MFI based on its marked differential expression between adipogenic differentiated and proliferating FAPs and significant correlation with adipogenic/fibrogenic biomarkers in skeletal muscle tissue. Experimental validation using the KLF5 inhibitor ML264 demonstrated significant suppression of adipogenic and fibrogenic markers in FAPs, while *in vivo* studies using an immobilization mouse model revealed that intramuscular ML264 administration effectively attenuated MFI progression, showing significant reduction in muscle atrophy and decrease in fibrotic area, and lower fatty infiltration compared to controls, collectively establishing KLF5 inhibition as a promising therapeutic strategy for MFI with its precise molecular mechanisms to be explored in-depth in our future study.

As MFI is well-established to be related to systemic status^[43]^, eQTL data from peripheral blood were also utilized to identify genes associated with MFI. Similarly, through various validation analyses, we identified three transcriptional regulatory genes (protein-coding) for MFI: C4A, C4B, and TRIM13. The C4A and C4B genes are part of the complement component 4 (C4) family, which plays a critical role in the immune system, particularly in the classical complement pathway^[43,44]^. These genes encode two isoforms of the C4 protein, which are involved in immune defense mechanisms, including the clearance of pathogens, immune complexes, and apoptotic cells. There was limited evidence about C4A with lipid metabolism but C4A was found to be associated with composition in terms of fat and protein percentage in milk production in animal study^[45]^. Besides, among human studies, both the C4A and C4B gene copy number variation were found to be significantly related to high-density lipoprotein in circulation^[46]^. In contrast, more studies have investigated C4B’s role in skeletal muscle homeostasis. One study demonstrated that aging increases C4B expression in skeletal muscle, delaying muscle progenitor cell proliferation and impairing functional recovery. Notably, C4B inactivation enhanced muscle regeneration both in vitro and *in vivo*, suggesting that age-related C4B upregulation may compromise muscle repair capacity^[47]^. The tripartite motif-containing protein 13 (TRIM13), a RING-type E3 ubiquitin ligase related to Calnexin/calreticulin cycle pathway and metabolism of proteins. One study found that TRIM13 could exacerbate lipid accumulation leading to atherosclerosis, and subsequent genetic deletion of TRIM13 protected against diet-induced atherosclerosis^[48]^. Furthermore, our study extends the understanding of MFI by integrating proteomic data in blood serum, ultimately identifying two circulating proteins that can influence MFI risk: CHRDL2 and HLA-E. These proteins are newly associated with MFI; however, CHRDL2 has been previously identified as promoting the activation of the adipogenesis-related downstream pathway BMP^[49]^. In contrast, HLA-E is related to inflammatory processes such as NK cell activity.

Following identification of MFI’s regulatory gene landscape, we investigated genetic associations with clinical phenotypes to enhance pathological understanding. MFI showed significant genetic correlations with key metabolic disorders (obesity, diabetes, cardiovascular diseases) and biomarkers (glucose, triglycerides, cholesterol), extending beyond localized muscle pathology to reflect systemic metabolic dysregulation. This provides new perspectives for metabolism-targeted interventions. Furthermore, MFI demonstrated associations with sarcopenia-related phenotypes (grip strength, muscle pain, fractures). Given these dual links to metabolic disease and sarcopenia, MFI represents a potential mechanistic bridge for studying their comorbidity, offering novel therapeutic targets for this patient population.

Given the associations between lipid metabolism-related phenotypes and MFI observed in previous studies and our research, we sought to pinpoint blood lipid metabolites that may causally influence the risk of MFI. This approach aimed to further validate the metabolic regulatory factors underlying MFI. Ultimately, we identified that Triacylglycerol (TAG) and PC significantly affected the risk of MFI. PC was the most abundant phospholipid of all mammalian cell types and subcellular organelles which comprises 40%–50% of total cellular phospholipids^[50]^. The phospholipids of skeletal muscle undergo remodeling in response to high-fat feeding^[51]^ and exercise^[52]^, etc. And if the PC synthesis or the molar ratio of PC/phosphatidylethanolamine in particular, was unbalanced as it was in subjects with obesity or reduced insulin sensitivity, the homeostasis of skeletal muscle would be disrupted especially for muscle insulin sensitivity and cellular calcium homeostasis^[51]^. TAG, which is a neutral lipid, has a structure in which three molecules of fatty acids are ester-bonded to one molecule of glycerol. The intramuscular TAG is stored within lipid droplets located in the cytoplasm of skeletal muscle cells^[53]^, proximal to mitochondria where the TAG-derived fatty acids are released for oxidation. It has been shown that intramuscular TAG played an important role in energy utilization both at rest and during physical activity in skeletal muscle^[54]^. Thus, PC and TAG may protect against and improve MFI by modulating insulin resistance and intramuscular mitochondrial fatty acid oxidation, respectively.

Despite this study conducting a comprehensive investigation of MFI risk genes across multiple levels (genomics, transcriptomics, and proteomics) and exploring their genetic associations with various metabolism- and musculoskeletal system-related phenotypes, certain limitations remain. Due to the objective constraint that MFI imaging data of a large population is currently available only in the UK Biobank, this study was unable to validate the GWAS findings using an independent replication cohort. Furthermore, although we experimentally screened regulatory targets in skeletal muscle and ultimately validated the effects of KLF5 on MFI in both cellular and animal models, the regulatory loci identified in whole blood – due to their complex associations with systemic conditions – were not experimentally validated in this study. This remains an important direction for our future research investigations.

Our integrated genomic/MRI analyses identified MFI-associated genes through GWAS and multi-omics approaches, revealing both known and novel regulators of lipid metabolism, adipogenesis, insulin resistance, and mitochondrial function. Notably, regulatory genes showed minimal overlap between muscle and blood tissues. Genetic correlations linked MFI to metabolic/musculoskeletal phenotypes, with lipidomics identifying 179 risk-associated metabolites. Experimental validation confirmed KLF5’s critical role in FAP fibro-adipogenesis and demonstrated its therapeutic potential in ameliorating MFI, muscle atrophy, and fibrosis in our immobilization model.

## Conclusion

Our comprehensive genomic and MRI analyses revealed MFI-associated genes via integrated GWAS and multi-omics approaches, identifying both established and novel regulators of lipid metabolism, adipogenesis, insulin resistance, and mitochondrial function. Notably, regulatory gene profiles demonstrated minimal concordance between muscle and blood tissues. Genetic correlation analyses further established connections between MFI and metabolic/musculoskeletal phenotype, while lipidomic profiling identified 179 risk-associated metabolites. Through experimental validation, we causally confirmed KLF5’s critical function in FAP fibro-adipogenesis and demonstrated its therapeutic efficacy in attenuating MFI, muscle atrophy, and fibrosis within our immobilization model.

## Data Availability

The data related to the main conclusions of the study have been included in the manuscript and supplementary materials, and the complete GWAS outcome data generated by the analysis will be provided when requested by the editor.

## Research registration unique identifying number (UIN)

In this study, no human individuals were collected. The data used are all from public databases such as UK Biobank.
